# South African clinical practice guidelines quality measured with complex and rapid appraisal instruments

**DOI:** 10.1186/s13104-016-2053-z

**Published:** 2016-04-27

**Authors:** Karen Grimmer, Shingai Machingaidze, Janine Dizon, Tamara Kredo, Quinette Louw, Taryn Young

**Affiliations:** International Centre for Allied Health Evidence (iCAHE), University of South Australia, City East Campus, P4-18 North Terrace, Adelaide, 5000 Australia; Department of Physiotherapy, Faculty of Medicine and Health Sciences, Stellenbosch University, Francie van Zijl Drive, Tygerberg, Cape Town, 7505 South Africa; European and Developing Countries Clinical Trial Partnership (EDCTP), Francie van Zijl Drive, Parow Valley, Cape Town, 7505 South Africa; South African Cochrane Centre (SACC), South African Medical Research Council, Francie van Zijl Drive, Parow Valley, Cape Town, 7505 South Africa; Centre for Evidence-Based Health Care (CEBHC), Faculty of Medicine and Health Sciences, Stellenbosch University, Francie van Zijl Drive, Tygerberg, Cape Town, 7505 South Africa; Center for Health Research and Movement Science, University of Santo Tomas, Espana, 1018 Manila, Philippines

**Keywords:** Clinical practice guidelines (CPGs), Rapid appraisal tool, iCAHE checklist, AGREE II, Complex appraisal tool, CPG quality, Reporting standards, Primary health care, South Africa

## Abstract

**Background:**

Critically appraising the quality of clinical practice guidelines (CPGs) is an essential element of evidence implementation. Critical appraisal considers the quality of CPG construction and reporting processes, and the credibility of the body of evidence underpinning recommendations. To date, the focus on CPG critical appraisal has come from researchers and evaluators, using complex appraisal instruments. Rapid critical appraisal is a relatively new approach for CPGs, which targets busy end-users such as service managers and clinicians. This paper compares the findings of two critical appraisal instruments: a rapid instrument (iCAHE) and a complex instrument (AGREE II). They were applied independently to 16 purposively-sampled, heterogeneous South African CPGs, written for eleven primary health care conditions/health areas. Overall scores, and scores in the two instruments’ common domains Scope and Purpose, Stakeholder involvement, Underlying evidence/Rigour of Development, Clarity), were compared using Pearson *r* correlations and intraclass correlation coefficients. CPGs with differences of 10 % or greater between scores were identified and reasons sought for such differences. The time taken to apply the instruments was recorded.

**Results:**

Both instruments identified the generally poor quality of the included CPGs, particularly in Rigour of Development. Correlation and agreement between instrument scores was moderate, and there were no overall significant score differences. Large differences in scores for some CPGs could be explained by differences in instrument construction and focus, and CPG construction. The iCAHE instrument was demonstrably quicker to use than the AGREE II instrument.

**Conclusions:**

Either instrument could be used with confidence to assess the quality of CPGs. The choice of appraisal instrument depends on the needs and time of end-users. Having an alternative (rapid) critical appraisal tool will potentially encourage busy end-users to identify and use good quality CPGs to inform practice decisions.

## Background

Over 20 years ago, Woolf [1–3] described clinical practice guidelines (CPGs) as ‘the new reality for medicine’. Research continues into how best to present this ‘new reality’ to end users in a way that will improve evidence uptake. Whilst there is no one internationally-agreed standard for developing CPGs [4–6], there is a general expectation that CPG recommendations should be transparently based on current best evidence [7–11].

End-users of CPGs are those who put CPG recommendations into operation, such as service managers and healthcare workers ‘at the coal face’. These people are rarely engaged in CPG writing [12], however they are usually well aware of the barriers to evidence-uptake [13–16]. These are consistently reported as lack of time, money, and knowledge [3, 13–16]. Thus when end-users choose a CPG, they need to be assured that it is of the best possible quality, and that it will efficiently assist them to provide quality care. Service managers and clinicians are busy people, and therefore to assist them in efficiently identifying and using quality CPGs, they require a time-efficient critical appraisal instrument that is comprehensive, simple, robust and efficient.

An Australian team at the International Centre for Allied Health Evidence (iCAHE), University of South Australia, developed and tested a 14 question binary-scored (yes = 1, no = 0) CPG appraisal instrument, designed specifically for busy end-users [17]. The iCAHE instrument was developed in partnership with service managers, policy-makers and clinicians, and incorporated their perceptions of important elements of CPG quality relevant to their settings. The iCAHE instrument contains 14 questions and provides one overall score (total out of 14). This scoring approach assumes equal weighting for each question, reflecting the views held by the end-users who assisted in its development.

The psychometric properties of the iCAHE instrument were established by comparison with AGREE II (Appraisal of Guideline Research and Evaluation), a complex CPG critical appraisal instrument [7, 18–20]. AGREE II is well-known internationally, and is recommended for assessing CPG quality by the South African Medical Journal [8, 9]. AGREE II has 23 statements grouped into six domains of Scope and Purpose; Stakeholder Involvement; Rigour of Development; Clarity of Presentation; Applicability; and Editorial Independence. Each statement is scored using a 1–7 scale, with 1 being no agreement and 7 being total agreement. The six domains in AGREE II are intended to be reported separately, and the scoring rubric is not designed to provide an overall quality score [18, 19].

The iCAHE and AGREE II instruments share four domains (Scope and Purpose, Stakeholder involvement, Underlying evidence/Rigour, Clarity). The iCAHE instrument also includes three domains not in AGREE II (currency, a summary of findings, and availability), whilst AGREE II includes two domains not in the iCAHE instrument (Applicability, and Independence) (see Table 1). The scores and utility of the iCAHE and AGREE II instruments were compared using six CPGs for mild traumatic brain injury [17]. Overall, the iCAHE and AGREE II scores correlated moderately well (Pearson *r* = 89 %). Depending on the complexity of CPG layout, the iCAHE instrument took between 5 and 10 min per-CPG to apply, whilst the AGREE II instrument scoring per-CPG per-tester took up to an hour.

**Table 1:** iCAHE questions mapped against AGREE II domains and their statements

	iCAHE	AGREE II
AGREE II domain 1: scope and purpose	Q13 Are the purpose and target users of the guideline stated?	Q1. The overall objectives of the guideline are specifically described
		Q2. The health questions covered by the guideline are specifically described
		Q3. The population to whom the guideline is meant to apply is specifically described
AGREE II domain 2: stakeholder involvement		Q6. The target users are clearly defined
	Q11. Are the developers clearly stated?	Q4. The guideline development group includes individuals from all relevant professional groups
	Q12. Does the qualifications and expertise of the guideline developers link with the purpose of the guideline and its end users?	Q5. The views and preferences of the target population have been sought
AGREE II domain 3: rigour of development	Q7. Does the guideline provide an outline of the strategy used to find underlying evidence?	Q7. Systematic methods were used to search for the evidence
	Q8. Does the guideline use a hierarchy to rank the quality of the underlying evidence?	Q8. The criteria for selecting the evidence are clearly described
	Q9. Does the guideline appraise the quality of the evidence which underpins its recommendations?	Q9. The strengths and limitations of the body of evidence are clearly described
	Q10. Does the guideline link the hierarchy and quality of underlying evidence to each recommendation?	Q10. The methods for formulating the recommendations are clearly described
		Q11. The health benefits, side effects and risks have been considered in formulating the recommendations
		Q12. There is an explicit link between the recommendations and the supporting evidence
		Q13. The guideline has been eternally reviewed by experts prior to its publication
		Q14. A procedure for updating the guideline is provided
iCAHE instrument domain: currency	Q4. Is there a date of completion available?	
	Q5. Does the guideline provide an anticipated review date?	
	Q6. Does the guideline provide dates for when literature was included?	
AGREE II domain 4: clarity of presentation	Q14. Is the guideline readable and easy to navigate?	Q15. The recommendations are specific and unambiguous
		Q16. The different options for management of the condition or health issues are clearly presented
		Q17. Key recommendations are easily identifiable
AGREE II domain 5: applicability		Q18. The guideline describes facilitators and barriers to its application
		Q19. The guideline provides advice and/or tools on how the recommendations can be put into practice
		Q20. The potential resources implications of applying the recommendations have been considered
		Q21. The guideline presents monitoring and/or auditing criteria
AGREE II domain 6: editorial independence		Q22. The views of the funding body have not influenced the content of the guideline
		Q23. Competing interests of guideline development group members have been recorded and addressed
iCAHE instrument domain: availability	Q1. Is the guideline readily available in full text?	
	Q2. Does the guideline provide a complete reference list?	
iCAHE instrument domain: summary	Q3. Does the guideline provide a summary of its recommendations?	

Adapted from Grimmer et al. [17]

The South African Guidelines Excellence (SAGE) is a project which aims to improve the quality of South African primary health care (PHC) CPGs. It is pursuing several research activities, namely identifying, and speaking with, key individuals and groups involved in PHC CPG writing and use in South Africa; determining the quality of current South African PHC CPGs and identifying ways to improve their quality; and building capacity in best practice CPG writing, implementation and evaluation in South African academics, clinicians and policy-makers [21]. The SAGE team recently reported on the quality of 16 purposively-sampled South African CPGs for priority PHC conditions, using AGREE II [22]. These CPGs comprised the most recent versions of seven disease-specific and four integrated multi-disease South African PHC CPGs (see Table 2, reproduced from Machingaidze et al. [22]). The dates of CPG publication ranged from 2002 to 2014. Overall, the quality domains of Rigour of Development, and Editorial Independence had the poorest scores, whilst Scope and Purpose, and Clarity of Presentation generally scored the best. The time taken to score each selected CPGs with AGREE II ranged between 45 and 60 min, depending on CPG layout, comprehensiveness and complexity.

**Table 2:** South African CPGs included in this analysis (reproduced from Machingaidze et al. [22])

Name	Short name	Publication year	Developer
*Disease specific guidelines*			
Clinical guidelines for the management of HIV and AIDS in adults and adolescents	Adult HIV	2010	National Department of Health
Guidelines for the management of HIV in children	Child HIV	2010	National Department of Health
Clinical guidelines: PMTCT (prevention of mother-to-child transmission)	PMTCT	2010	National Department of Health
National tuberculosis management guidelines	Adult TB	2014	National Department of Health
Guidelines for the management of tuberculosis in children	Child TB	2013	National Department of Health
Malaria prevention guidelines	Malaria prevention	2011	National Department of Health
Malaria treatment guidelines	Malaria treatment	2010	National Department of Health
*Combination guidelines*			
Standard treatment guidelines and essential medicines list for South Africa	EDL	2008	National Department of Health
Integrated management of childhood illnesses	IMCI	2002	National Department of Health
Guidelines for maternity care in South Africa	Maternal	2007	National Department of Health
Primary care 101	PC101	2013	UCT Lung Institute/National Department of Health
*Guidelines by professional societies*			
Guideline for the management of acute asthma in adults: 2013 update	Adult asthma	2013	South African Thoracic Society
Guideline for the management of acute asthma in children: 2013 update	Child asthma	2013	South African Thoracic Society
Guideline for the management of chronic obstructive pulmonary disease—2011 update	COPD	2011	South African Thoracic Society
South African hypertension guideline 2011	Hypertension	2011	Southern African Hypertension Society
The 2012 SEMDSA guideline for the management of type 2 diabetes (Revised)	Type II diabetes	2012	Society for endocrinology, metabolism and diabetes of South Africa

AGREE II was developed for, and has been largely used by, researchers and CPG developers, thus its use may present challenges for time-constrained end-users who have to assess CPG quality by themselves. The iCAHE instrument could be a viable alternative to AGREE II when a rapid overview of CPG quality is required. This paper describes how the iCAHE instrument compares to the AGREE II instrument on a larger set of heterogeneous CPGs.

## Methods

### Data set

The same 16 purposively-selected South African PHC CPGs reported by Machingaidze et al. [22] were assessed using the iCAHE instrument, and the scores from the two instruments were compared.

### Scoring

The iCAHE instrument was applied by one independent experienced tester whose level of experience was similar to that of the testers who applied the AGREE II instrument [22].

### Data management

To facilitate comparison between instrument scores for each CPG, a percent of possible total (overall) score was calculated for the iCAHE instrument and also from the AGREE II instrument. This approach was previously used when initially validating the iCAHE instrument against AGREE II [17], even though a total AGREE II score is not calculated from the AGREE II domain rubric [18, 19]. To calculate one percent total score, the individual item responses for all AGREE II statements were applied to the scoring rubric, using a minimum possible score of 23 (calculated as 23 items*1), and a maximum possible score of 171, calculated as 23 items*7. This score was then reported as a percentage of the possible total.

### Analysis

Correlation between instrument scores was reported as Pearson correlation coefficients *(*Pearson *r)*. Significance of instrument score differences was determined using *p* values from single factor analysis of variance (ANOVA) models, and intraclass correlation coefficients (ICC_(2,1)_) were calculated from the mean square outputs of these ANOVA models. The ICC_(2,1)_ calculation assumed that the testers were similar to those who might use the instruments in other situations. CPGs with instrument score differences of >10 % (where positive differences favoured the iCAHE instrument) were identified. The two datasets were:

1. The % total iCAHE scores and the % total AGREE II scores for each CPG, using all items in each instrument (23 AGREE II statements, 14 iCAHE questions).
2. The % total scores for only the items in the instruments’ common domains (Scope and Purpose, Stakeholder involvement, Rigour of development, Clarity of Presentation). This involved eight iCAHE questions and 17 AGREE II statements. The same process of calculating total AGREE II scores was used as described in the *Data management* paragraph, however the denominators were 8 (8*1) for iCAHE and 119 (17 items*7) for AGREE II.

The time spent critically appraising the iCAHE instrument was recorded for each CPG, and compared with the time reported by Machingaidze et al. [22].

## Results

### Overall CPG quality

**Fig. 1:**
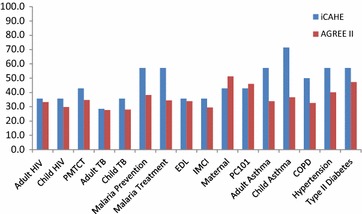
Analysis 1 findings: comparison of % of total scores per CPG, including all questions (iCAHE instrument) and statements (AGREE II)

**Fig. 2:**
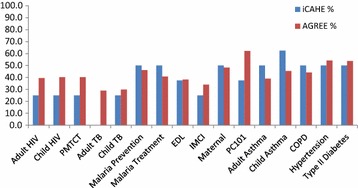
Analysis 2 findings: comparison of % of total scores per CPG for the common domains only

Irrespective of whether the iCAHE or AGREE II instrument was used, or the number of questions/statements compared, the overall quality of reporting in the South African PC CPGs was generally poor (See Table 3; Figs. 1 and 2).

**Table 3:** CPG scores for iCAHE questions mapped against AGREE II domains

Domains	iCAHE questions	Disease specific guidelines							Combination guidelines				Guidelines by professional societies				
		Adult HIV	Child HIV	PMTCT	Adult TB	Child TB	Malaria prevention	Malaria treatment	EDL	IMCI	Maternal	PC101	Adult asthma	Child asthma	COPD	Hypertension	Type II diabetes
AGREE II domain 1: scope and purpose	Q13	1	1	1	0	1	1	1	1	1	1	1	0	0	1	1	1
AGREE II domain 2: stakeholder involvement	Q11	0	0	0	0	0	1	1	1	0	1	1	1	1	1	1	1
	Q12	0	0	0	0	0	1	1	0	0	1	0	1	1	1	1	1
AGREE II domain 3: rigour of development	Q7	0	0	0	0	0	0	0	0	0	0	0	0	1	0	0	0
	Q8	0	0	0	0	0	0	0	0	0	0	0	1	1	0	0	0
	Q9	0	0	0	0	0	0	0	0	0	0	0	0	0	0	0	0
	Q10	0	0	0	0	0	0	0	0	0	0	0	0	0	0	0	0
New iCAHE domain: currency	Q4	1	1	1	1	1	1	1	1	1	1	1	1	1	0	1	1
	Q5	0	0	0	0	0	0	0	0	0	0	0	0	0	0	0	0
	Q6	0	0	0	0	0	0	0	0	0	0	0	0	1	0	0	0
AGREE II domain 4: clarity of presentation	Q14	1	1	1	0	1	1	1	1	1	1	1	1	1	1	1	1
New iCAHE domain: summary	Q3	1	1	1	1	1	1	1	0	1	0	1	1	1	1	1	1
New iCAHE domain: availability	Q1	1	1	1	1	1	1	1	1	1	1	1	1	1	1	1	1
	Q2	0	0	1	1	0	1	1	0	0	0	0	1	1	1	1	1
Guideline % score^a^		36	36	43	29	36	57	57	36	36	43	43	57	71	50	57	57

*1* Yes (criterion was addressed), *0* No (criterion not addressed)

^a^Domain percentage scores are calculated as the total number of yes questions divided by the total number of possible questions and converted into a percentage

Table 4 reports the findings from analyses 1 and 2. Comparing analysis 1 with 2, there was an improved correlation between instrument scores for analysis 2, as well as a stronger ICC_(2,1)_ score (with a lower, but not significant, p value). There were no significant percentage of score differences overall, from either analysis 1 or 2. However from analysis 1, the large score differences all favoured the iCAHE instrument (see Fig. 1), whilst from analysis 2, the large score differences mostly favoured the AGREE II instrument (see Fig. 2).

**Table 4:** *Pearson r* correlation coefficients, ICC_(2,1)_ values for agreement and CPGs with disagreements >10 %, listed by order of size of disagreement for analyses 1 and 2

	Pearson r correlation coefficient	Agreement expressed as ICC_(2,1)_ values	CPGs with disagreements > 10 % between instruments	Size of disagreement (-ve values in favour of AGREE II) (%)
Analysis 1: using all statements in both instruments	*0.39*	*ICC* = *0.06 (p* = *0.39)*	*(6 of 16)*	
			Child asthma	34.8
			Adult asthma	23.3
			Malaria treatment	22.7
			Malaria prevention	18.9
			COPD	17.4
			Hypertension	17.1
Analysis 2: using only the statements in the common domains between the instruments	*0.61*	*ICC* = *0.49 (p* = *0.07)*	*(7 of 16)*	
			Child asthma	17.1
			Adult asthma	10.9
			Adult HIV	−14.5
			Child HIV	−15.3
			PMTCT	−15.3
			PC101	−24.7
			Adult TB	−29.0

The time to use the iCAHE instrument was 3–5 min per CPG. This mirrored earlier findings on the utility of the iCAHE instrument [17].

## Discussion

This study compare findings from a complex CPG critical appraisal instrument (AGREE II) with a rapid appraisal instrument (iCAHE), on a sizeable sample of heterogeneous country-specific PHC CPGs. Scoring CPG quality is an essential element of evidence implementation [10, 11, 13–16]. Unless end-users have confidence in the quality of the evidence underpinning CPG recommendations, they are unlikely to adopt them. CPGs offer ready access to a ‘one-stop-shop’ for current best evidence-summaries [1–3]. Irrespective of which critical appraisal instrument was used (rapid or complex), we identified consistent concerns relating to the quality of the selected South African PHC CPGs, particularly in Rigour of Development. This is a similar finding to other studies evaluating South African CPG quality [8, 9].

Analysis 1, which compared the per-CPG total scores derived from the 23 AGREE statements, and the 14 iCAHE questions, demonstrated the modesty of both correlation and agreement. This was attributed to the variability in number and intent, in the two instruments’ items. For instance, whilst there were four common domains between instruments, the iCAHE questions included additional domains of Currency, Availability, and Summary, whilst the AGREE II instrument included additional domains of Applicability and Editorial Independence. Comparing differences in total scores, all six CPGs with large percent differences (>10 %) favoured the iCAHE instrument.

Analysis 2, which compared data from just the four shared domains in the iCAHE and AGREE II instruments, showed improved correlation and agreement, but identified different CPGs with large score differences (with only two of the seven highlighted CPGs favouring the iCAHE instrument). This suggests that the between-instrument differences in the number of statements/questions in the common domains possibly influenced the scoring (8 iCAHE questions in four domains, 17 AGREE II statements in the same four domains). This potentially weighted the overall score in favour of AGREE II.

The shorter time taken to score CPG quality using iCAHE instrument compared with AGREE II reflects the smaller number of items, as well as the utility of the binary-scored iCAHE instrument, where no subjectivity in interpretation is required. In comparison, Machingaidze et al. [22] reported that the AGREE II scores took as much as 10 times longer to compile per CPG, as its use required personal judgement identify a score from 1 to 7 for each statement, and then the application of a scoring rubric per domain. As previously reported [17], this potentially introduces uncertainty in critical appraisal.

## Conclusions

Both appraisal instruments provide standard valid and reliable frameworks for assessment of CPG quality, albeit oriented for different end users. Thus either instrument could be used with confidence to assess the quality of a CPG, and the choice of instrument would depend on the purpose of appraisal, available time and whether additional personnel were available to apply the AGREE II scoring requirements. Having an alternative (rapid) critical appraisal tool will potentially encourage busy end-users who may not currently use complex tools such as AGREE II, to identify good quality CPGs to inform practice and policy decisions.

## Abbreviations

CPG(s): clinical practice guideline(s)
iCAHE: International Centre for Allied Health Evidence, University of South Australia
AGREE II: Appraisal of Guideline Research and Evaluation
SAGE: South African Guidelines Excellence
PHC: primary health care
ANOVA: analysis of Variance
ICC: intraclass correlation coefficient
